# Treating depression in patients with borderline personality disorder: clinical clues on the use of antidepressants

**DOI:** 10.1186/s12991-024-00507-z

**Published:** 2024-05-30

**Authors:** Carmine Tomasetti, G. Autullo, A. Ballerini, A. de Bartolomeis, B. Dell’Osso, A. Fiorentini, F. Tonioni, V. Villari, D. De Berardis

**Affiliations:** 1 Department of Mental Health, Alzheimer Center of Giulianova, Hospital “Maria SS dello Splendore”, ASL Teramo, Giulianova (TE), Italy; 2 Psychiatry and Psychology Institute, Catholic University of Sacred Heart of Rome, Rome, Italy; 3 Psychiatry Unit, Department of Health Science, University of Florence, Largo Brambilla 3, Florence, 50134 Italy; 4 Laboratory of Molecular and Translational Psychiatry and Unit of Treatment Resistant Psychosis, Section of Psychiatry, Department of Neuroscience, Reproductive Science and Dentistry, University of Naples “Federico II”, Naples, Italy; 5 Department of Mental Health, Department of Biomedical and Clinical Sciences Luigi Sacco, University of Milan, Milan, Italy; 6 Department of Neurosciences and Mental Health, Ca’ Granda Ospedale Maggiore Policlinico, Fondazione Istituto di Ricerca e Cura a Carattere Scientifico (IRCCS), University of Milan, Milan, Italy; 7 Psychiatric Emergency Service, Department of Neuroscience and Mental Health, A.O.U. “Città della Salute e della Scienza”, Turin, Italy; 8 Department of Mental Health, Mental Health Center of Giulianova, ASL Teramo, Teramo, Italy

**Keywords:** Serotonin antagonist/Reuptake inhibitors, Comorbidity, Psychiatric disorders, Trazodone, Neurobiology, Emotion

## Abstract

Personality disorders (PD) are described as enduring patterns of markedly deviant and pervasive inner experiences and behaviors, with onset in adolescence, which lead to severe distress or impairment. Patients suffering from major depressive disorder (MDD) display higher rates of comorbidity with personality disorders, often complicating the treatment, and worsening the outcomes. Borderline personality disorder (BPD) is the most common of PD and is frequently associated with MDD, with which shares several features. The most part of research agrees on the fact that comorbid BPD in MDD patients quite doubles the poor response to treatments. Moreover, no treatment strategy stands out currently to emerge as more effective in these cases, thus urging the call for the need of new approaches. Herein, we revise the current literature on BPD, its neurobiology and comorbidity with MDD, as well as the more recent treatment strategies used. Then, based on its pharmacology, we propose a possible role of trazodone as a valuable tool to approach comorbid BPD-MDD.

## Introduction

Major Depressive Disorder (MDD) is a leading contributor to global burden of disease, being considered as a major cause of disability worldwide, with approximately 3.8% of population affected and over 700.000 people dying of suicide every year [1]. Despite multiple treatment strategies have been developed, MDD remains a serious challenge for psychiatrists, since approximate 30% of patients do not adequately respond to therapies. The largest MDD trial, the so-called STAR*D (Sequenced Treatment Alternatives to Relieve Depression), demonstrated that, even after 4 consequential steps of treatment, the cumulative remission rate reached 67% after 14 months [2].

Since MDD is a heterogeneous disorder, multiple reasons have been put forward to support these high rates of treatment resistance: misdiagnosis (e.g., bipolar depression, or mixed states); comorbid substance use; untreated medical conditions (e.g., dysthyroidism); undiagnosed underlying traumata (i.e., post-traumatic stress disorder); cognitive impairment (i.e., neurocognitive disorders) [3].

In addition to the above-mentioned contributors, a large body of evidence points out the essential role of underpinning and/or understated personality disorders (PD) in the scarce responsivity of MDD to treatments. PDs comorbidity has been recognized in almost half of MDD patients in different meta-analyses [4, 5]. Indeed, the pervasive symptoms of PDs, such as low self-esteem, self-criticism, mood instability, feeling of emptiness or hopelessness, suicidal thoughts or behaviors, may all represent substantial contributors to worsen or complicate depression, or even to make depressive symptoms persistent and resistant [6]. Several studies have examined the relationships amongst personality disorders traits and the quality, the severity, and the outcomes of MDD [7, 8]. Personality disorders have been correlated to earlier onset of MDD, to specific subtypes of depression (melancholic vs. non melancholic depression) [5], as well as to severer symptoms (i.e., suicidal behaviors, self-harming, impaired cognition), and poorer outcomes (e.g., greater resistance to pharmacological and non-pharmacological treatments) [9]. Thus, the frequent association between PDs and MDD poses the classical question whether came first the chicken or the egg, since from a psychological point of view some predisposing risk factors may be associated to both the conditions [10]. Moreover, given this entangled relationship, a diagnostic issue should be considered, when assessing a patient with MDD; but, more important, a complete revision of therapeutic approaches to the treatment of depression should be contemplated, based on the possible influence of underlying preponderant personality traits in depressed patients.

In the next sections, we will consider the impact of the most devastating PD, the Borderline Personality Disorder, on MDD, and we will discuss the possible revision of classical antidepressant treatments in the light of an integrated neurobiological-psychological approach to MDD therapy.

## The influence of comorbid Borderline Personality Disorder on Major Depressive Disorder

Borderline Personality Disorder (BPD) is described in the Diagnostic and Statistical Manual of Mental Disorders, fifth edition, text revision 2022, (DSM-5-TR) [11] as a “pervasive pattern of instability of interpersonal relationships, self-image, and affects, and marked impulsivity, beginning by early adulthood and present in a variety of contexts”. Sensation of abandonment, unstable relationships, identity disturbances, impulsivity, recurrent suicidal behaviors, affective instability, feelings of emptiness, anger and, occasionally, transient dissociative or psychotic symptoms during periods of distress may be all features of BPD. All these features can be grouped in three main categories (“factors”, according to DSM-5-TR): disturbed relatedness, behavioral dysregulation, and affective dysregulation; all of these being strongly correlated with each others, thus representing a unique construct, although with broad and pleiotropic manifestations [12]. BPD is the most common PD, with a reported prevalence of 10% in all psychiatric outpatients [13], and 5.9% in non-clinical population [14]. Moreover, the most part of BPD patients seem not to access psychiatric care, but they represent a significant part of primary care patients, since BPD has been described as four-times more prevalent amongst general practitioners’ costumers as compared to general population [15].

Several studies have reported a high frequency of co-occurrence between BPD and MDD, since 83–85% of BPD patients have been described to suffer from MDD episodes, with high recurrences [16–18]. Moreover, amongst PDs, BPD seems to have the highest correlation with both genetic and environmental risk factors of MDD [19].

Manifold studies have tried to dig up the intrinsic connections between BPD and MDD, and just as many theories and models have been developed, with the precise aim to improve diagnosis and therapy of these disorders, above all when comorbid. Personality has been characteristically described as a dynamic construct of two main components: temperament, the biologically-based structure of emotion regulation, and character, which instead is modulated by social relations [20]. According to the Five Factors Model (FFM), personality traits may be taxonomically subdivided in five principal characteristics, the so-called Big Fives: neuroticism, extraversion, conscientiousness, agreeableness, and openness to experience. Given the hierarchical relationships amongst these factors, they may be further grouped in two big clusters: positive emotionality and negative emotionality [21]. There is increasing evidence that, although personality traits have solid genetical and biological bases, they are not rigid constructs, but may be dynamically modulated by development and life experiences [22]. Psychologically, MDD is characterized by a substantial deficiency in positive emotionality, with a prevalence of negative emotions, such as sadness, guilt, shame, anhedonia, depressed mood, and numbness (i.e., the inability of feeling feelings) [23]. However, it is not rare that MDD patients may manifest irritability, anger, hostility, which are all factors often correlated to self-harming or suicide [24, 25]. By contrast, the whole symptomatologic cortege of BPD is mainly hinged on impulsivity traits, with emotional dysregulation, anger, dyscontrol, dysphoria, self-harming, and hostility [26]. Nevertheless, some typical features of BPD may resemble those seen in MDD patients, such as the feeling of emptiness, sadness, loneliness of hopelessness [27].

Therefore, BPD may add an “impulsivity color” to MDD symptoms framework, when the two disturbs manifest comorbidly. Different studies, indeed, reported that BPD patients experiencing MDD show increased levels of anger, fear, and hostility [28], as well as they manifest considerably higher impulsivity than MDD-only patients [29]. Moreover, BPD patients diagnosed with MDD tend to describe their depressive symptoms as more serious as compared to MDD-only patients, even severer than those objectively assessed by physicians [30, 31].

Notably, BPD has been demonstrated to show significant comorbidity also with Bipolar Disorder (BD). Indeed, by analyzing data from the National Epidemiologic Survey on Alcohol and Related Conditions (NESARC), McDermid et al. found that the lifetime prevalence of BPD was about 29% in BD type I and 24% in BD type II, and that comorbid BPD-BD had severer presentation as compared to BD alone [32]. Successive studies confirmed that BPD may represent a risk factor for BD, and remarked the negative impact of BPD in BD, such as the higher prevalence of suicidality and the treatment-resistance [33]. Moreover, about 40% of MDD-diagnosed patients have been reported to have a history of subthreshold hypomania symptoms, this subgroup showing earlier onset and more episodes of depression, as well as more comorbidities as compared to non-hypomanic patients [34]. Last, MDD episodes where psychomotor agitation and racing thoughts are found (the so-called “agitated depression”) have been robustly associated to mixed states, unfavorably predicting the emergence of suicidal ideation and contraindicating antidepressant therapy [35]. This tight intermingling between hypomanic symptoms, MDD, and BPD may challenge the dichotomic vision of unipolar-bipolar depression, suggesting a more comprehensive “mood spectrum” diagnostic approach [36].

Thus, it is possible that the inherent “bipolarity” of BPD may colorize MDD with unstable emotional traits, lending this disorder an increased resistance to treatments, as compared to MDD alone. The Collaborative Longitudinal Personality Disorders Study (CLPS) analyzed the longitudinal course of BPD patients, as compared to other PDs and MDD-only patients [37]. Amongst the other interesting results, CLPS reported that 80% of BPD patients assessed had MDD, and that MDD-only patients showed a remission rate dramatically faster (80% by 1 year) as compared to BPD patients (30% by one year), thus indicating how much BPD comorbidity may influence MDD outcome. These results have been confirmed by the National Epidemiological Study of Alcoholism and Related Disorders, which reported that BPD was the major predictor of persistent MDD [38]. The McLean Study of Adult Development (MSAD) further demonstrated that, when BPD and MDD coexist, the remission of MDD strictly depends upon the remission of BPD symptoms [39], thus confirming a previous landmark study, in which an improvement in MDD symptoms was found when treating BPD symptoms, but not vice versa [40]. However, the mainstay treatment for MDD, antidepressant drugs, have been demonstrated to promote only partial responses in MDD-BPD patients [39]. Thus, there is a peculiar tendence to poly-treat BPD patients, which have been described to averagely take three-to-five medications, an over-prescription that could be only reduced by a BPD-specific psychotherapy regimens [41]. Also, non-pharmacological treatments, such as electroconvulsive therapy (ECT) and transcranial magnetic stimulation (TMS) have demonstrated partial efficacy in treating comorbid MDD and BPD [42, 43]. Therefore, it appears rather obvious that the treatment of MDD in course of BPD relies on an efficacious BPD treatment. Hence, some specific psychotherapy regimens, such as Dialectical behavioral therapy (DBT) have demonstrated a good efficacy in improve MDD symptoms by improving BPD [44].

So, is there a biological basis on which the BPD-induced MDD treatment-resistance relies? And, in the light of this possible underlying basis, should it be possible to reconsider a targeted pharmacological approach to help reducing the impact of BPD on MDD?

## Digging in the deep: the neurobiological bases of BPD and the underpinnings of comorbid MDD

Although BPD has been classically envisioned as a complex multifactorial disorder, in which environmental risk factors (e.g., traumata, abuses, neglect) seems to be preponderantly responsible for its development [45], increasing evidence has been pointing out the essential role of the genetic factors underlying the specific personality traits at the basis of the disorder. Indeed, twin studies have demonstrated that BPD has a heritability ranging from 46 to 69% [46, 47]. Recently, different genome-wide association studies (GWAS) have been performed, in order to study genetic association of the “Big Five” factors of FFM with PDs in general population. BPD was found closely associated with personality traits of neuroticism and openness [48], and, more interestingly, it was reported to share positive genetic correlations with MDD, Bipolar Disorder and Schizophrenia [49]. As above-mentioned, BPD represents a unique construct intermingling specific personality traits, such as disturbed relatedness, behavioral and emotional dysregulation. However, despite the manifold researches stating the large heritability of BPD, only a few genetic studies exist, which tried to correlate these personality traits to specific gene dysfunctions. As previously mentioned, Witt and collaborators found a significant overlap of BPD-associated genes with those associated to MDD, Bipolar Disorder and Schizophrenia [49]. Two genes reached genome-wide significance: dihydropyrimidine dehydrogenase (DPYD) and Plakophilin-4 (PKP4). DPYD is implicated in pyrimidine metabolism and contains a binding site for the micro-RNA miR-137, which has been found associated to Schizophrenia [50]. PKP4 is involved in the regulation of cell adhesion and cytoskeletal modifications, which have been substantially implicated in cell junction deficits associated to MDD [51]. Previously, Lubke et al. have described a specifical association of BPD with the serine incorporator 5 gene (SERINC5), which seems to have a peculiar role in myelination, and has been involved in the development of psychiatric disorders characterized by lack of social interactions [52, 53]. Finally, a genome-wide linkage study found a significant association of BPD features with chromosome 9 loci, which have been significantly associated also to Bipolar Disorder and Schizophrenia [54].

Given the essential role of environment in BPD development, it is not surprising that a large number of studies have reported abnormalities in BPD in epigenetic modifications, which are considered the “portal” through which environment may impact gene expression changes, via DNA methylation, histone deacetylation and non-coding RNA silencing [55]. Altered methylation of specific genes, such as dopamine D2 receptors, serotonin 3A receptors, glucocorticoid receptors and brain-derived neurotrophic factor (BDNF) receptors have been all associated to BPD [56–59]. It is interesting to note that these alterations may be directly correlated to the severity of childhood abuse in BPD patients [60], as well as to the intensity of depressive symptoms, and may be reinstated by specific psychotherapy regimens [61].

As already discussed, the core symptoms of BPD rely on a substantial emotional dysregulation. Different studies reported altered emotional interoception in BPD patients, the so-called alexithymia (i.e., “no words for emotions”): while their amygdaloid system highly responds to negative emotions, they have a blunted self-report of the experienced emotions [62]. This may be due to an altered regulatory control of amygdala-based emotion system: indeed, BPD patients have been described to have altered connections between prefrontal cortex and amygdala, thus probably having an impaired top-down emotional modulation [63]. Moreover, both substance use, and dissociative episodes have been reported to dampen the hyperactive emotional responses in BPD patients, thereby explaining the frequent comorbidity of BPD with substance use disorder (SUD), as well as the higher frequency of dissociative experiences in BPD patients [64, 65]. Interestingly, altered amygdaloid responses and neuroplasticity have been demonstrated in MDD patients [66]. Moreover, a particular kind of treatment-resistant depression, called “dissociative depression”, has been characterized as frequent in younger patients with childhood traumata, and is defined by the higher frequency in dissociative episodes, as well as by its chronicity, mood instability, and often by comorbid BPD [67]. Finally, SUD is frequently diagnosed also in MDD patients, and some etiopathogenetic models propose that substances may help depressed patients to cope with their altered affective states [68].

Besides emotional dysregulation, as previously mentioned, BPD patients experience an essential disrupted relatedness, with interpersonal sensitivity leading to social difficulties.

Several studies have associated BPD social dysfunctionality to altered opioidergic and neuropeptidergic neurotransmission. Primarily, opioidergic neurotransmission is correlated in humans with pain responses. Increasing evidence suggests that µ-opioid receptors may mediate both sensory and affective dimension of pain, in different cerebral regions [69]; moreover, pain may be literally perceived in social exclusion and rejection by means of µ-opioid mediation in brain [70, 71]. BPD patients have been demonstrated to possess a lower sensitivity to acute pain, but a heightened sensitivity to chronic pain [72, 73]. This altered sensitivity to pain may be essentially due to an abnormal µ-opioid transmission: indeed, BPD patients have been demonstrated to possess a greater number of cortical µ-opioid receptors, probably due to a scarce baseline opioidergic transmission, with altered and enhanced compensatory opioid responses to acute stimuli [74]. Besides its primary role in pain responses modulation, µ-opioid neurotransmission has been associated to the right development of attachment behaviors in mammals [75, 76]. Interestingly, altered µ-opioid gene expression has been found in adolescents prone to develop MDD reactions to social rejection life events [77].

Oxytocinergic neurotransmission has been also found abnormal in BPD patients, which were reported to have lower levels of oxytocin as compared to healthy individuals, these levels being correlated with childhood traumata and disrupted attachment [78, 79]. Moreover, while in healthy subjects oxytocin administration usually enhances social behaviors, in BPD patients it may provoke counterintuitive aversive behaviors, especially correlated to history of childhood traumata [80]. Last, genetic alterations in oxytocin receptors have been directly correlated to the development of BPD in abused children [81, 82]. The increasing evidence of a substantial role of oxytocin in the etiopathogenesis of MDD, as well as in its possible treatments, represents a further bridge between BPD and MDD [83, 84].

Monoaminergic neurotransmission has been implicated in personality since long ago. Particularly, personality dimensions as described by Cloninger, and later by the FFM, may be directly linked to dopaminergic, serotonergic, and noradrenergic neurotransmissions [85–87].

Dopamine dysfunctions, for example, have been associated to three specific dimensions of BPD: impulsivity, emotional dysregulation, and cognitive impairment [88]. Specific genetic polymorphisms of the dopamine transporter gene (DAT1) have been peculiarly associated with increased risk of BPD in MDD patients [89]. Moreover, the same polymorphism has been associated to angry-impulsive traits in comorbid BPD-MDD patients [90]. On the other hand, both serotonin transporter (5HTT) and serotonin A1 receptor (5HT1A) genes have been associated with BPD [91, 92]. Specifically, 5HT1A gene alterations have ben correlated to abnormal amygdala structure and emotional responses in BPD-MDD comorbid patients [91]. Serotonin alterations seem to be tightly correlated to the “impulsivity color” of MDD, when comorbid with BPD, as well as with an increased risk of suicide [93, 94]. Recent studies demonstrated that serotonin and dopamine neurotransmissions closely interact in defining the personality traits underlying BPD, and the simultaneous presence of both dysfunctions may interplay in favoring the risk of BPD development [95]. Norepinephrine, along with cortisol, has been associated to dissociative responses in BPD [96].

## Targeting depression in BPD: clinical clues on the use of antidepressants. Focus on trazodone

BPD patients, with their pleiotropic symptomatologic manifestations, represent a huge burden for health systems. In fact, as above mentioned, BPD is frequently associated to coexisting psychiatric disorders, above all MDD, anxiety, substance use, and it is as much as frequently misdiagnosed [97]. Due to their comorbidities, as well as to their over-endorsement of symptoms, BPD patients often tend to self-medicate (even with substances) or to access primary cares, where they are not often understood and well-treated [98]. Although BPD patients have been described to have good chances to remit over the long period [99], during the trajectory of the disorder, they have frequent relapses and serious outbursts, which lead to multiple accesses to mental health services for specialized cares or hospitalizations [100].

All the most recent guidelines for the treatment of BPD seems to agree on the fact that a specific regimen of psychotherapy should be the first line treatment, whereas medications should be used with caution for intense and disruptive symptoms during decompensation acuity, and only for the shortest possible time [101–104]. However, while European guidelines—which include NICE (National Institute for Health Care and Excellence) ones—suggest to pharmacologically treat only comorbid disorders in BPD [103, 104], APA (American Psychiatric Association) and WFSBP (World Federation of Societies of Biological Psychiatry) suggest using specific classes of medications to treat specific symptom domains [101, 102]: thus, antidepressants should be be used to treat emotional dysregulation and impulsivity, similarly to mood stabilizers, while antipsychotics should be used for dissociative and cognitive-perceptual symptoms.

Although scarce evidence exists on the efficacy of antidepressant treatments in BPD, SSRIs (Selective Serotonin Reuptake Inhibitors) are currently the most prescribed medications [105]. The most part of RCTs examining the efficacy of antidepressants in BPD are outdated, and they have not been replicated since 2010. The main antidepressants for which data are available in the treatment of BPD are: fluoxetine, fluvoxamine, sertraline, amitriptyline, phenelzine, venlafaxine, mianserin. A Cochrane review [106] reported that antidepressants had no significant effects on the overall BPD severity; no beneficial effects were noticed on impulsivity, as well as on suicidal behaviors, whereas a worsening of suicidal ideation was noticed with fluoxetine; affective instability was slightly ameliorated by fluvoxamine, while no significant effects were noticed for self-harming, feeling of emptiness, anger; the only significant effects on depression were found with amitriptyline.

Similar results were obtained in a comparative meta-analysis by Vita et al. [107], with a documented, although slight, effect of antidepressants only on affective dysregulation.

Significant results have been achieved on MDD comorbid to BPD when antidepressants were combined to mentalizing psychotherapies (DBT, IPT [Interpersonal Psychotherapy]) [108, 109].

It is worth noting that all the antidepressant drugs chosen to be tested in BPD patients, as above described, were selected based on their well-documented efficacy on MDD, which is primarily due to serotonergic effects (i.e., serotonin re-uptake inhibition), with generally scarce impact on other neurotransmitters, such as dopamine or norepinephrine [110]. On the other hand, the most significant effects in reported RCTs were obtained by means of antidepressant drugs that involved more than the sole serotonin neurotransmission, such as phenelzine, amitriptyline and fluoxetine, or even by combined treatments (e.g. fluoxetine plus olanzapine), which were able to control—although slightly—the core affective/emotional instability, which is the typical signature of BPD [106].

As above described, BPD core depressive symptoms have been hypothesized to involve multiple neurobiological substrates, such as opioidergic and oxytocinergic neurotransmission, and specific monoamine receptors, such as dopamine D2 and serotonin 2A receptors, which interplay with each others to generate the symptoms of comorbid MDD-BPD. Thus, a more targeted pharmacological approach might help to relieve, if only partially, depressive symptoms in BPD.

In this light, a revision of “old” antidepressant treatments, relying on the enhancement of their possible efficacy, based on their peculiar pharmacodynamic properties, might represent a valuable approach. According to this view, trazodone may be a useful tool to address the unmet needs of MDD in BPD.

The history itself of trazodone appears intriguing, if envisioned in the light of the abovementioned neurobiological underpinnings of BPD. Indeed, it is a triazolopyridine derivative, which was developed in 1960s in Italy based on the “mental pain” hypothesis of MDD, correlating depressive states to altered pain interoception [111]. Along with nefazodone, trazodone represents the prototype of the so-called serotonin antagonist/reuptake inhibitor antidepressants (SARIs). It is a powerful antagonist at 5HT2A serotonin receptors, which are bound already at low doses, together with alpha1- and alpha2- adrenergic receptors and H1 histamine receptors, thus exerting potent anxiolytic and sedative/hypnotic effects at these doses [112]. Trazodone also weakly binds the serotonin transporter (SERT), 5HT2B and 5HT2C serotonin receptors, even if it is not clear if it acts as a full agonist, a partial agonist or an antagonist at these last receptors [112]. Another peculiar characteristic is the strong binding to 5HT1A serotonin receptors, where it acts as a partial agonist with high intrinsic activity [113]. Moreover, trazodone has an active metabolite, the meta-chlorophenylpiperazine (mCPP), which is known to exert pro-serotonergic psychoactive functions similar to fenfluramine and MDMA (“ecstasy”), in addition to being a well-recognized agonist to multiple serotonin receptors (e.g., 5HT1A, 5HT1B, 5HT1D, 5HT2A, 5HT2B, 5HT2C, 5HT3, and 5HT7 receptors) [114, 115]. Thus, trazodone shapes up to be a peculiar multimodal antidepressant, which may exert differential functions at different doses. In fact, the progressive recruitment of serotonin receptors—in particular 5HT2A and 5HT1A—at incremental dosages has been described to exert incremental antidepressant effects by means of multiple—and not completely understood-- mechanisms: (1) 5HT1A receptors activation may mediate some neurotrophic factors’ genes expression, which has been associated to antidepressant actions; (2) 5HT1A receptors may progressively inhibit glutamate release from cortical pyramidal neurons, whose hyperactivity has been implicated in cognitive symptoms of MDD; (3) 5HT2A and 5HT2C serotonin receptors blockade has been associated to the increase in dopamine and noradrenaline cortical release, which are complementary to serotonin in relieving depressive symptoms [112].

Currently, trazodone is marketed in three different formulations: immediate release (IR), prolonged release (PR), and once-a-day extended release (OAD). Trazodone IR has a rapid plasma peak (1 h) and a short half-life (6.6 h); trazodone PR has a slower plasma peak (4 h) and a longer half-life (12 h), and trazodone OAD shows a plateau plasma level for the entire day, with longer antidepressant concentration as compared to the other formulations [113]. A large amount of data supports the fact that trazodone has similar efficacy to all the other antidepressants when compared to placebo [116]. Moreover, the OAD formulation has been described to grant a higher antidepressant efficacy than a placebo with a once-a-day administration, with side effects comparable to other antidepressants [117]. Finally, trazodone displays high tolerability, even when administered in patients with comorbid clinical conditions, thus granting a safety profile in poly-pharmaco-treated patients [118].

Several characteristics of trazodone may let lean forward its valuable use in comorbid BPD-MDD patients.

As previously described, the concurrent blockade of 5HT2A/2C receptors and of SERT, the partial agonist activity at 5HT1A receptors, and the antagonism at 5HT7 receptors may boost the antidepressant action of trazodone by increasing serotonin postsynaptic action and the subsequent disinhibition of dopamine and noradrenaline cortical release, together with glutamate-modulated neurotrophic factors’ gene expression [112, 119]. Indeed, some studies have described the rapid onset of trazodone antidepressant effects. Sheehan et al. [117] demonstrated that trazodone OAD (150-225 mg/day) may induce a substantial reduction in depressive symptoms within a week of treatment, and that these effects may persist until 56 weeks. Fagiolini et al. [120] reported a faster antidepressant response (within 7 days) in patients treated with trazodone OAD (150 mg/die) as compared to venlafaxine XR (75 mg/die). This faster antidepressant effects of trazodone were not only exerted, as mainly expected, on the sleep component of depressive symptoms, but also on the cognitive aspects of depression [121].

The rapid antidepressant action of trazodone could be really useful during the fast emotional outbursts of BPD patients, which often lead to hospitalization. Peculiarly, this fast action seems not to be accompanied by a heightened risk of suicidal behaviors, even in high-risk patients treated with trazodone [122].

Trazodone has been demonstrated to exert antinociceptive effects even at low dosages, possibility via a µ-opioid receptors-mediated mechanism [123, 124]. These properties may be helpful in manage the altered pain interoception of BPD patients—their “mental pain”—, as well as in treating their susceptibility to auto-medication with analgesics or substances. Indeed, diverse studies provided evidence of a good efficacy of trazodone in the treatment of alcohol, benzodiazepines, and opioid abuse [125, 126].

The off-label clinical use of trazodone as hypnotic is well-established [126]. Some BPD patients have been described to have particularly disrupted sleep, with frequent nightmares, which in turn have been correlated to an increased risk of dissociative experiences and suicidal behaviors [127], above all if related to childhood traumatic events [128]. Trazodone has been demonstrated to be particularly effective in improving the quality of sleeping and reducing nightmares in post-traumatic stress disorders-affected war veterans [129].

## Conclusions

BPD is a devastating personality disorder, with multiple symptomatologic presentations, and often comorbid with mood disorders, particularly with MDD, thereby making it substantially treatment resistant. SSRIs have been demonstrated to be scarcely efficacious on BPD-MDD patients. However, the neurobiological underpinnings of BPD may suggest that a more targeted antidepressant approach may helpful in relieving BPD-MDD coexisting symptoms. Since its multimodal action on serotonin, dopamine, noradrenalin, opioid and glutamate neurotransmissions, as well as its incremental effectiveness, trazodone seems to embody all the characteristics which may make it a clinical valuable tool to be used in BPD-MDD patients. More specifically designed studies are warranted to corroborate these clinical clues.

## Data Availability

No datasets were generated or analysed during the current study.
